# Closed and High-Quality Bacterial Genome Sequences of the Oligo-Mouse-Microbiota Community

**DOI:** 10.1128/MRA.01396-20

**Published:** 2021-04-29

**Authors:** Quentin Lamy-Besnier, Romain Koszul, Laurent Debarbieux, Martial Marbouty

**Affiliations:** a Bacteriophage, Bacterium, Host Laboratory, Department of Microbiology, Institut Pasteur, Paris, France; b Unité Régulation Spatiale des Génomes, Institut Pasteur, CNRS, UMR 3525, Paris, France; c Université Paris Descartes, Paris, France; Indiana University, Bloomington

## Abstract

The Oligo-Mouse-Microbiota (OMM^12^) gnotobiotic murine model is an increasingly popular model in microbiota studies. However, following Illumina and PacBio sequencing, the genomes of the 12 strains could not be closed. Here, we used genomic chromosome conformation capture (Hi-C) data to reorganize, close, and improve the quality of these 12 genomes.

## ANNOUNCEMENT

The Oligo-Mouse-Microbiota (OMM^12^) is a murine bacterial synthetic community (12 strains) introduced in axenic mice. The resulting gnotobiotic murine model has been increasingly used for diverse gut microbiota studies across several animal facilities (1–4). This bacterial consortium comprises members from the 5 major phyla naturally present in mice microbiota and reconstitutes its main functionalities, such as metabolism and colonization resistance against pathogens (5).

Despite two rounds of sequencing using PacBio and Illumina technologies (6, 7), one-half of the genomes currently accessible remain made of 2 to 20 contigs (Table 1). In particular, the genome of Bacteroides caecimuris, the most abundant bacterium of the OMM^12^ consortium, consists of 19 contigs (5). Improving scaffolding would facilitate a number of downstream analyses, such as prophage prediction.

**TABLE 1:** Description and accession numbers of the OMM^12^ genomes^*a*^

Strain	Genome size (bp)	Previous no. of contigs^*b*^	No. of genes	DSM no.	Accession no.	Reads (%)^*c*^
Akkermansia muciniphila YL44	2,737,357	1	2,717	26127	CP065322	2.8
Acutalibacter muris KB18	3,802,913	1	4,271	26090	CP065321	0.002
Bifidobacterium animalis YL2	2,021,936	2	1,740	26074	CP065311	0.0007
Bacteroides caecimuris I48	4,800,606	19	4,322	26085	CP065319	55.8
Blautia coccoides YL58	5,128,582	1	5,351	26115	CP065312	20.5
Enterocloster clostridioformis YL32	7,157,610	16	7,955	26114	CP065314	4.9
Clostridium innocuum I46	4,452,146	1	4,966	26113	CP065320	5.6
Enterococcus faecalis KB1	3,025,655	1	2,919	32036	CP065317	0.1
Flavonifractor plautii YL31	3,813,715	5	4,049	26117	CP065315	0.38
Limosilactobacillus reuteri I49	2,063,624	3	1,958	32035	CP065318	0.99
Muribaculum intestinale YL27	3,307,069	1	2,957	28989	CP065316	4.5
Turicimonas muris YL45	2,887,949	20	2,753	26109	CP065313	0.089

a Raw data can be accessed at SRX9907181.

b Garzetti at al. (6).

c Unmapped reads = 4.34 %.

We used genomic chromosome conformation capture (Hi-C), a technique that uses chemical fixation (formaldehyde) to *in vivo* cross-link nearby DNA sequences, digestion with restriction enzymes, DNA extremities refilling with biotinylated nucleotides, and finally, proximity ligation, DNA extraction, and deep sequencing. The relative ligation frequencies between nonadjacent DNA segments reflect the average three-dimensional (3D) organization of the genome of interest (8) and also, because of the polymer nature of DNA, the relative distance separating them along the chromosome. The latter property has therefore been exploited to bridge scaffolding gaps resulting, for instance, from the presence of repeated elements (9). Hi-C scaffolding is now commonly used in sequencing projects of large eukaryotic genomes but applies to bacteria as well (10, 11).

A Hi-C metagenomic protocol for mammalian gut samples developed in our laboratory (12) was applied to fecal pellets from the OMM^12^ mice bred at Institut Pasteur (protocol 20.173 approved by the veterinary staff and under authorization APAFIS 26874 by the national ethics committee). Libraries were prepared using streptavidin beads to capture biotinylated ligation junction as described previously (13) and sequenced with an Illumina NextSeq 550 system to generate a total of 101,182,905 reads of 35 bp. The quality of the reads was first verified with FastQC (http://www.bioinformatics.babraham.ac.uk/projects/fastqc). Then, for each bacterium, the python library hicstuff (https://github.com/koszullab/hicstuff) was used to generate Hi-C contact maps using the most recent OMM^12^ genomes (6). Contigs were reorganized (i.e., scaffolded) based on their relative contact frequencies (14). When necessary, preexisting contigs were split and rearranged to better fit the expected 3D structure, based on the contact data. In order for users to detect junctions between contigs, contigs were separated by 10 × *N* in the final fasta file. We successfully closed the genomes of the 12 strains except for Flavonifractor plautii, where the position of a 70,760-bp region could not be assigned into the main scaffold. The genome of *F. plautii* therefore remains split in 2 scaffolds. An automatic annotation was then performed using RAST (15). The access to a single scaffold of high quality will greatly improve the accuracy of genomic analyses performed by the community of users of the OMM^12^ gnotobiotic mice.

### Data availability.

The 12 reassembled and closed genome sequences of the OMM^12^ bacteria as well as the FastQ reads have been deposited under the accession numbers provided in Table 1 under the BioProject number PRJNA680355.
